# A non-canonical multisubunit RNA polymerase encoded by a giant bacteriophage

**DOI:** 10.1093/nar/gkv1095

**Published:** 2015-10-20

**Authors:** Maria Yakunina, Tatyana Artamonova, Sergei Borukhov, Kira S. Makarova, Konstantin Severinov, Leonid Minakhin

**Affiliations:** 1 Peter the Great St. Petersburg Polytechnic University, St. Petersburg, 195251, Russia; 2 Department of Molecular Biology and Biochemistry, Waksman Institute, Rutgers, The State University of New Jersey, Piscataway, NJ 08854–8020, USA; 3 Rowan University School of Osteopathic Medicine, Stratford, NJ 08084–1501, USA; 4 National Center for Biotechnology Information NLM, National Institutes of Health Bethesda, MD 20894, USA; 5 Skolkovo Institute of Science and Technology, Skolkovo, 143026, Russia

## Abstract

The infection of *Pseudomonas aeruginosa* by the giant bacteriophage phiKZ is resistant to host RNA polymerase (RNAP) inhibitor rifampicin. phiKZ encodes two sets of polypeptides that are distantly related to fragments of the two largest subunits of cellular multisubunit RNAPs. Polypeptides of one set are encoded by middle phage genes and are found in the phiKZ virions. Polypeptides of the second set are encoded by early phage genes and are absent from virions. Here, we report isolation of a five-subunit RNAP from phiKZ-infected cells. Four subunits of this enzyme are cellular RNAP subunits homologs of the non-virion set; the fifth subunit is a protein of unknown function. *In vitro*, this complex initiates transcription from late phiKZ promoters in rifampicin-resistant manner. Thus, this enzyme is a non-virion phiKZ RNAP responsible for transcription of late phage genes. The phiKZ RNAP lacks identifiable assembly and promoter specificity subunits/factors characteristic for eukaryal, archaeal and bacterial RNAPs and thus provides a unique model for comparative analysis of the mechanism, regulation and evolution of this important class of enzymes.

## INTRODUCTION

Transcription is the first step of gene expression and the main target of regulation. All transcription enzymes, the DNA-dependent RNA polymerases (RNAPs), catalyze template-dependent RNA synthesis via a common two-metal mechanism (1,2). Based on subunit composition, conserved amino acid motifs, and three-dimensional structures, RNAPs can be divided into two unrelated families. One family comprises single-subunit enzymes (ssRNAPs), and is part of a larger superfamily of ‘right-handed’ DNA and RNA polymerases (3). Enzymes of the ssRNAP family are involved in transcription of phage (notably, the prototypical *Escherichia coli* T7 phage), mitochondrial and chloroplast genomes. The second family comprises multisubunit enzymes (msRNAPs) that transcribe genes in cells from all three domains of life: bacteria, archaea and eukaryotes. Together with eukaryotic RNA-dependent RNAPs involved in RNAi silencing, the msRNAP family forms a superfamily of ‘two-barrel’ RNAPs (4). In all known msRNAPs, the conserved double-ψ β-barrel (DPBB) domains are formed by parts of the two largest subunits. The DPBB domain of the largest subunit (β’ in bacteria) contains a metal-binding motif DxDGD with three invariant aspartates coordinating the catalytic Mg^2+^ (1–3). The DPBB of the second-largest subunit (β in bacteria) donates additional invariant amino acids necessary for catalysis (4). On their own, the largest RNAP subunits are catalytically inactive (5). Additional universally conserved msRNAP subunits (a dimer of α subunits and the ω subunit in bacteria) assemble the large catalytic subunits into a functional complex (6–10). In eukaryotes, archaea and some bacteria, various auxiliary subunits associate with the basic β’βα_2_ω core (11,12)

The RNAP core is catalytically proficient but is unable to specifically initiate transcription from promoters (13). Despite the high level of sequence and structural similarity (14,15), transcription initiation by eukaryal and archaeal RNAPs on the one side, and bacterial RNAPs on the other side, is accomplished by completely different, evolutionary unrelated mechanisms. In bacteria, binding of one of the several σ subunits endows the core enzyme with the ability to specifically recognize and locally melt promoter DNA, positioning the RNAP catalytic center at the transcription start point and allowing transcription initiation (13,16,17). In eukaryotes and archaea transcription initiation is much more complex, involving a large set of general transcription factors that recruit RNAP to promoters, induce promoter melting and determine selection of the transcription start point (11,18).

Bacteriophages are ultimate parasites that rely on resources of their bacterial hosts for propagation. Most known phages with double-stranded DNA genomes use the host RNAP throughout the infection, modulating its function to ensure ordered expression of viral genes (19,20). Some phages encode their own RNAPs that transcribe specific sets of viral genes. The T7-like bacteriophages utilize host RNAP at the beginning of infection to transcribe the phage's own RNAP gene and then rely on phage-encoded enzyme to transcribe middle and late phage genes (21). The N4-like phages evolved a ‘reverse’ strategy that involves injection of a virion-encapsulated RNAP, vRNAP, along with viral DNA into the host cell (22,23). The vRNAP transcribes early genes, which include two genes for another, non-virion RNAP (24). This enzyme transcribes middle phage genes, while late transcription is carried out by host RNAP holoenzyme modified by the phage (25,26).

All known phage-encoded transcription enzymes belong to the ssRNAP family. The only possible exception may be an unusual multisubunit RNAP that has been isolated from cells infected by the *Bacillus subtilis* PBS2 phage (27,28). However, the lack of the PBS2 genomic sequence makes it impossible to determine the relationship of this enzyme to other phage or cellular RNAPs. Members of a group of giant phages related to the *Pseudomonas aeruginosa* phiKZ phage encode two sets of proteins homologous to N- and C-terminal fragments of the largest msRNAP subunits (29–31). Four proteins from one set are present in phiKZ virions and may comprise vRNAP that could be injected in the host together with viral DNA and perform early phage gene transcription, as is the case during the N4 infection. The phiKZ infection is resistant to host bacterial RNAP inhibitor rifampicin (30), suggesting that the virus relies on its own transcription machinery for the entire infection process. The second set of fragmented RNAP-like proteins may therefore form an additional, non-virion phage RNAP that could transcribe middle or late phage genes. The putative phage RNAPs should have a unique subunit composition, since the phiKZ genome does not encode recognizable subunits responsible for largest RNAP subunits assembly (α, ω) or promoter recognition (σ).

In this paper, we describe purification and initial biochemical characterization of non-virion RNAP from phiKZ-infected cells. The enzyme consists of five polypeptides, the products of early phage genes. Two subunits correspond to N- and C-terminal parts of β’ subunit and two other subunits correspond to N- and C-terminal parts of β subunit of msRNAP. The fifth subunit has no homologs in public databases except for counterparts encoded in the genomes of giant phages of the phiKZ family. The phiKZ nvRNAP lacks subunits with similarities to known msRNAP assembly subunits (α and ω in bacteria) or promoter specificity subunits (σ in bacteria, general transcription factors in eukaryotes). Yet, the nvRNAP specifically transcribes from late phiKZ promoters *in vitro*. Further analysis of assembly and promoter recognition by nvRNAP will thus provide a new vintage point for comparisons of msRNAPs function.

## MATERIALS AND METHODS

### Bacteriophages, bacterial strains and growth conditions

The strain of *P. aeruginosa* PAO1 wild type and *P. aeruginosa* PAO1 *rpoA::strep* strain encoding a strep-tag attached to the RNAP α subunit (32) were kindly provided by Dr Rob Lavigne from Laboratory of Gene Technology, KU Leuven, Belgium. Phage phiKZ was obtained from Dr V.N. Krylov from Russian Academy of Sciences Institute of Bioorganic Chemistry, Moscow, Russia. Тhe *P. aeruginosa* strains were grown in LB medium at 37°C. High-titer phage phiKZ preparations were prepared from lysed infected PAO1 cultures and purified by centrifugation at 10 000 g for 10 min. To prepare infected cells for RNAP purification an overnight PAO1 culture was diluted 1:100 in 1 l of fresh LB medium and, after reaching OD_600_ of 0.6 was infected with phiKZ at multiplicity of infection of 10. Cells were allowed to grow and infection to continue until indicated time points and terminated by the addition of 100 mg/ml chloramphenicol and rapid cooling on an ice water bath. Cells were harvested by centrifugation (6000 g for 10 min), flash-frozen and stored at -80°C until use. The efficiency of infection was checked by determining the number of remaining colony forming units in aliquots of infected cultures collected 10 min post infection. Only cultures that contained less than 20% of surviving cells were used for further processing.

### Purification of *P. aeruginosa* RNAP

*P. aeruginosa* RNAP σ^70^-holoenzyme was purified from PAO1 *rpoA::strep* cells. Briefly, 2 g of *P. aeruginosa rpoA::strep* strain biomass was disrupted by sonication in 10 ml buffer A (40 mM Tris-HCl, pH 8.0, 5% glycerol, 0.5 mM EDTA, 0.2 mg/ml PMSF, 2 mM β-mercaptoethanol) containing 200 mM NaCl followed by centrifugation at 15 000 g for 30 min. The cleared lysate was loaded onto 1 ml Strep-Tactin Superflow high capacity cartridge (IBA). The column was washed with 20 ml buffer A. The RNAP was eluted with buffer A containing 2.5 mM desthiobiotin. Eluted protein fractions were concentrated by ultrafiltration (Amicon Ultra-4 Centrifugal Filter Unit with Ultracel-100 membrane, EMD Millipore) and loaded onto Superose 6 10/300 (GE Healthcare) gel filtration column equilibrated with buffer A containing 200 mM NaCl. The fractions with the RNAP σ^70^-holoenzyme were pooled, diluted 2-fold with buffer A and concentrated. Glycerol was added up to 50% to the sample for storage at −20°C.

### Purification of nvRNAP

Twenty grams infected PAO1 biomass harvested 20–30 min after phiKZ infection was disrupted by sonication in 100 ml of buffer A containing 50 mM NaCl followed by centrifugation at 15 000 g for 30 min. An 8% Polyethylenimine P (pH 8.0) solution was added with stirring to the cleared lysate to the final concentration of 0.8%. The resulting suspension was incubated on ice for 30 min and centrifuged at 10 000 g for 15 min. The pellet was washed once by resuspension in buffer A with 0.1 M NaCl, centrifuged, and resuspended in buffer A with 0.3 M NaCl. Eluted proteins were precipitated by adding ammonium sulfate to 67% saturation, dissolved in buffer A without NaCl and loaded onto a 5 ml HiTrap Heparin HP sepharose column (GE Healthcare) equilibrated with buffer A with 0.1 M NaCl. The column was washed with buffer A with 0.3 M NaCl, and nvRNAP was eluted with buffer A with 0.6 M NaCl. This fraction was diluted with buffer A to a final 0.05 M NaCl concentration and applied onto a 1 ml HiTrap DEAE FF column (GE Healthcare) equilibrated with the same buffer. In 0.15–0.35 M NaCl linear gradient, nvRNAP was eluted at 0.17–0.24 M NaCl concentrations. The nvRNAP-containing fractions were pooled, concentrated by ultrafiltration (Amicon Ultra-4 Centrifugal Filter Unit with Ultracel-30 membrane, EMD Millipore) and loaded onto a Superdex 200 Increase 10/300 (GE Healthcare) gel filtration column equilibrated with buffer A containing 200 mM NaCl. As a final purification step, the combined nvRNAP fractions eluted from the Superdex 200 column were diluted 2-fold with buffer A and applied to a MonoQ HR 5/5 column (GE Healthcare). Bound proteins were eluted with a linear 0.15–0.25 M NaCl gradient in buffer A. The nvRNAP was eluted from the column at 0.18–0.19 M NaCl. The nvRNAP subunits containing fractions were concentrated to a final concentration 1 mg/ml, then glycerol was added up to 50% to the sample for storage at −20°C.

### Native gel electrophoresis

One microgram of nvRNAP was resolved by a native PAGE on a 4–15% gradient Phast gel (Pharmacia) using native gel strips. A single band was revealed by Coomassie blue staining. To determine the protein composition of this band, it was excised from the native gel and the gel chip was placed into a well of a 4–12% gradient SDS polyacrylamide gel (Novex^®^, Life Technologies) followed by the addition of 5–8 μl of Laemmli loading buffer. After electrophoresis, proteins were detected using Coomassie blue staining.

### Mass spectrometric identification of proteins after SDS-PAGE

Protein bands of interest were manually excised from the Coomassie-stained SDS gels. Individual slices were prepared for mass-spectrometry by ‘in-gel’ trypsin digestion at 37°C for 4 h (33) or, for rapid identification in chromatographic fractions, at 55°C for 30 min. 0.4 μl of eluted tryptic peptides were mixed on the target plate with 0.4 μl 2,5-dihydroxybenzoic acid (DHB)(Aldrich) diluted to 20 μg/ml in 50% acetonitril and 0.1% TFA and air dried. Mass-spectrometric analysis was conducted on FTICR-MS 9.4 Tesla (Varian 902-MS) in MALDI mode. Proteins were identified using the Mascot (www.matrixscience.com) program against the NCBI database. The search criteria for both Mascot searches were as follows: trypsin digestion with one missed cleavage allowed, the maximum peptide mass tolerance was ±5 ppm.

### *In vitro* transcription

Multiple-round run-off transcription reactions were performed in 10 μl of transcription buffer (40 mM Tris-HCl, pH 8.0, 10 mM MgCl_2_, 5 mM DTT) with or without 40 mM KCl (for *P. aeruginosa* RNAP or nvRNAP, correspondingly). Reaction mixtures contained 200 nM nvRNAP or *P. aeruginosa* RNAP and 50–100 nM of PCR-amplified DNA fragments containing corresponding promoters. Reactions were incubated for 10 min at 37°C, followed by the addition of ATP, CTP and GTP (0.3 mM each), 30 μM UTP and 3 μCi [α-^32^P]UTP (3000Ci/mmol). Where indicated, rifampicin was added to the final concentration 40 μg/ml. Reactions proceeded for 10 min at 37°C and were terminated by the addition of an equal volume of urea-formamide loading buffer. Reaction products were resolved on 8% (w/v) denaturing polyacrylamide gels and visualized using a PhosphorImager (Molecular Dynamics).

Abortive transcription initiation reactions were set at the same general conditions as run-off transcription reactions. Reactions were incubated for 10 min at 37°C, followed by the addition of 100–500 μM of initiating RNA dinucleotides specified by the -1/+1 positions of promoters studied (CpA for the T7 A1 promoter and UpG for late phiKZ promoters used) and 30 μM [α-^32^P] UTP (30 Ci/mmol). The reactions were allowed to proceed for 10 min at 37°C and terminated by the addition of an equal volume of urea-formamide loading buffer. Abortive initiation reaction products were resolved on 20% (w/v) denaturing polyacrylamide gels and visualized using a PhosphorImager (Molecular Dynamics).

### Primer extension reactions

*In vivo* primer extension reaction was done essentially as described elsewhere (30). *In vitro* transcription reaction for subsequent primer extension analysis contained, in 100 μl transcription buffer for nvRNAP, 1 mM NTPs mix, 30 nM nvRNAP and 50–100 nM of PCR fragments containing phiKZ promoters. Reactions were allowed to proceed for 30 min at 37°C followed by DNase I treatment, acid phenol (pH 6.0)/chloroform treatment and ethanol precipitation. Purified RNA was dissolved in RNase-free water and used as a template in primer extension reactions. RNA was reverse-transcribed with M-MLV reverse transcriptase (Life Technologies) in the presence of 10 pmol of γ-^32^P end-labeled primer according to the manufacturer protocol. The reactions were precipitated with ethanol and dissolved in urea-formamide loading buffer. As markers, DNA sequencing reactions with PCR-amplified phiKZ promoter fragments and the same end-labeled primers used for the primer extension reaction were performed. The reaction products were resolved on 6–8% (w/v) polyacrylamide sequencing gels and visualized using a PhosphorImager (Molecular Dynamics).

### Sequence analysis

Sequence similarity searches were performed using PSI-BLAST and TBLASTN programs (34) against NCBI non-redundant (NR) and whole genome shotgun databases with inclusion threshold E-value = 0.01 and no composition based statistics correction. We used HHpred server (http://toolkit.tuebingen.mpg.de/hhpred) for pairwise alignment of phiKZ RNAP subunits and their homologs with respective β’ and β subunits of *Thermus thermophulis* RNAP, pdb: 2a6h (35). Conserved sequence features identified for all RNAP homologs previously (14,15) were mapped on phiKZ nvRNAP sequences manually based on sequence and structures similarities with >90% HHpred probability. For better accuracy of identification and better visualization of the selected regions multiple sequence alignments of nvRNAP subunits and msRNAP conserved regions were done using AlignX program from Vector NTI software (Termo Fisher Scientific) followed by manual correction based on the PSI-BLAST and HHpred results.

## RESULTS

### Purification and subunit composition of phiKZ-encoded RNAP

**Figure 1.**
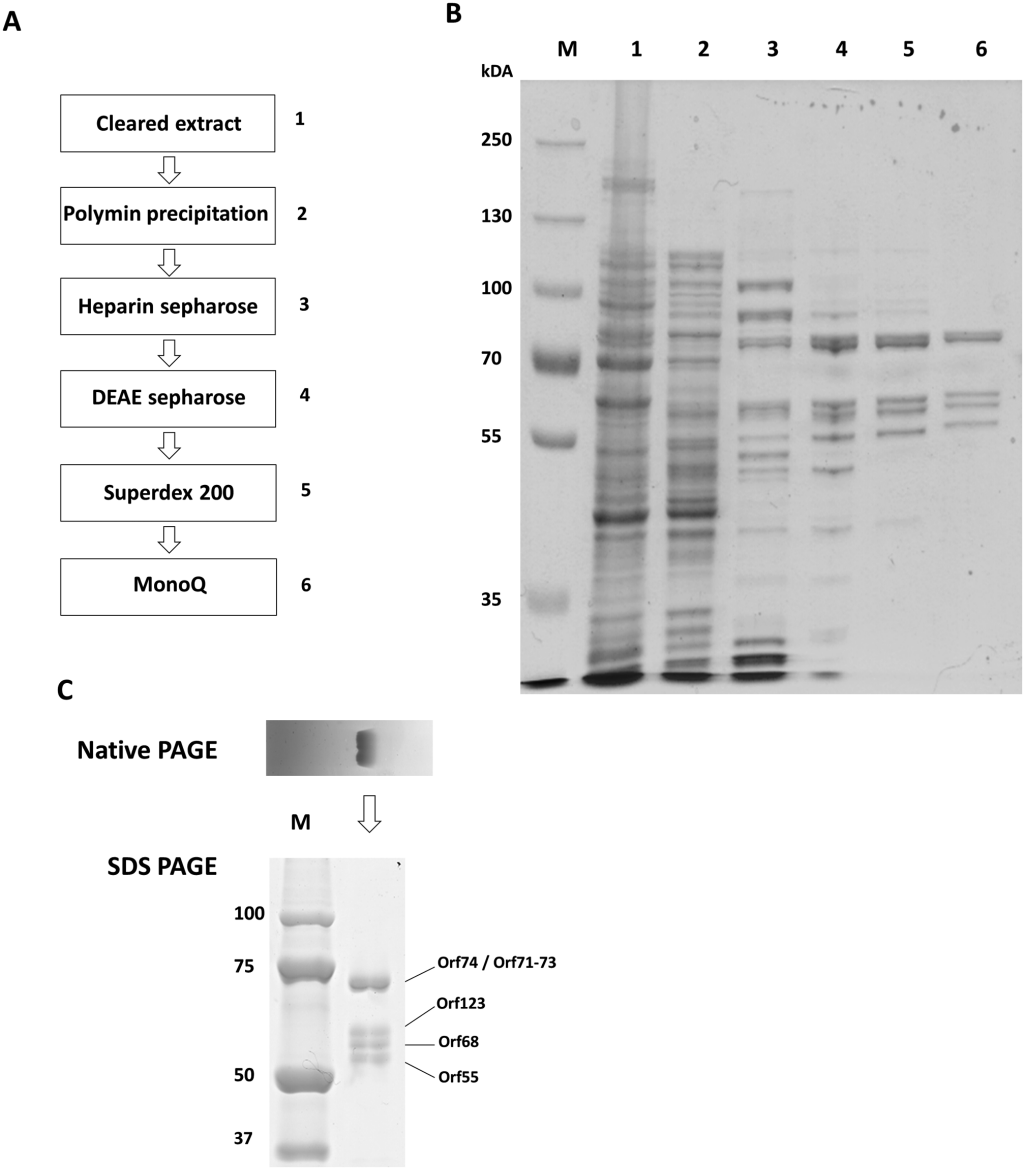
Purification of phiKZ-encoded nvRNAP. (**A**) The sequence of steps used to purify nvRNAP is shown. (**B**) А Coomassie-stained SDS gel of fractions containing gp55 and gp74 during nvRNAP purification with the gel lane numbers corresponding to the numbers of steps from panel А. (**C**) Native PAGE analysis of nvRNAP from the final MonoQ purification step (upper panel) and subunit composition of the native PAGE band (lower panel). Individual protein bands were identified by mass-spectrometry.

Purification of phage RNAPs from phiKZ-infected *P. aeruginosa* cells was undertaken. Cells were collected 30 min post infection, at the onset of late phage transcription (30). The procedure was based on the standard purification of bacterial RNAP (36). The presence of phage RNAP-like proteins was monitored by mass-spectrometric analysis of protein bands after SDS-PAGE separation of components of individual fractions. After polyethylenimine P precipitation, a standard first step during bacterial RNAP purification (36), an extract of the pellet with a buffer containing 0.3 M NaCl was found to contain non-virion RNAP-like proteins gp55 and gp74. Host RNAP β and β’ and proteins of the putative virion phage RNAP-like were not detected in this extract. We decided to concentrate on this sample with a goal of purifying gp55, gp74 and any associated proteins. A sequence of chromatographic steps (including heparin-sepharose, DEAE-sepharose, Superdex 200 and MonoQ columns) was elaborated with electrophoretic and mass-spectrometric monitoring of gp55 and gp74 in the fractions (Figure 1A). As a result, a single peak eluting from a MonoQ column was obtained. SDS-PAGE revealed the presence of five polypeptides in apparently stoichiometric amounts in peak fractions (Figure 1B, lane 6). Native PAGE of the material from peak fraction revealed a single band. SDS-PAGE analysis of polypeptide content of the native gel band revealed all five polypeptides (Figure 1C), which, therefore, form a stable complex. Mass-spectrometric analysis demonstrated that the complex consists of the five polypeptides: gp55 (56 kDa), gp68 (59 kDa), gp71–73 (74 kDa), gp74 (77 kDa) and gp123 (63 kDa), hereafter referred to as phiKZ non-virion (nv) RNAP.

### Sequence analysis of nvRNAP protein sequences

**Figure 2.**
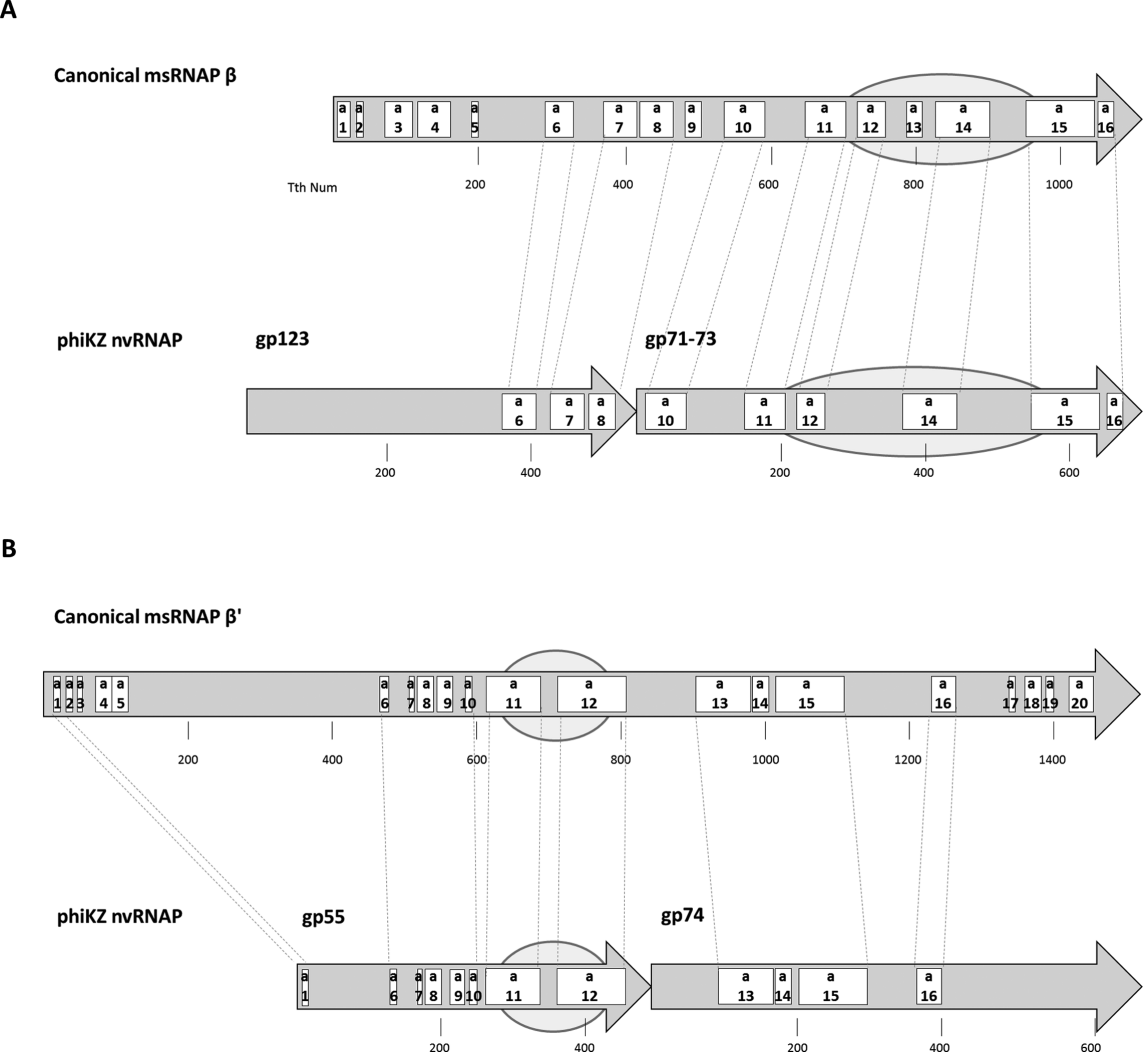
Conserved sequence and structural regions of the phiKZ nvRNAP subunits. The second largest (β in bacteria) and the largest (β’ in bacteria) cellular multisubunit RNAPs subunits are shown as arrows in panels **A** and **B**, respectively. Evolutionarily conserved sequence regions are labeled according to the Lane and Darst nomenclature: a1-a16 for β and a1-a20 for β’; the size of subunits and the positions of conserved regions are shown for *Thermus thermophilus* RNAP as a representative bacterial enzyme. Below the β and β’ subunits schemes ihe phiKZ nvRNAP subunits are shown, with sequence regions similar to corresponding msRNAP subunits identified and connected to each other by thin dotted lines. The alignments of conserved regions are presented in Supplementary Figure S1. The DPBB domains that form msRNAP catalytic centers are marked as ovals.

Sequence and structural features of msRNAP in all three domains of life are very well studied. In particular they have been thoroughly analyzed and described in detail by Lane and Darst (14,15). We used HHpred and PSI-BLAST programs to search for sequence similarity between nvRNAP and msRNAP subunits and map sequence and structural features identified in the Lane and Darst work (14) to nvRNAP subunit sequences (Figure 2, Supplementary Figure S1). This analysis revealed that gp55 and gp74 together correspond to msRNAP largest (bacterial β’) subunits covering sequence motifs a1 and a6 to a15 and including the catalytic DPBB domain (Figure 2, Supplementary Figure S1). The gp71–73 subunit corresponds to the C-terminal half of msRNAP second largest (β in bacteria) subunits with conservation of structural motifs a10-a16 and includes another DPBB domain required for msRNAP catalytic center formation. The gp123 protein appears to be a highly diverged homolog of the N-terminal half of the second largest (β in bacteria) msRNAP subunits with only few conserved motifs detectable (Figure 2, Supplementary Figure S1). The fifth protein of the nvRNAP, gp68, has no similarity to either known RNAP subunits or any other known protein family. Its counterparts, however, are identified in all other phiKZ-like genomes, suggesting it plays an essential role in the nvRNAP complex.

### *In vitro* transcription by phiKZ RNAP from late promoters

**Figure 3.**
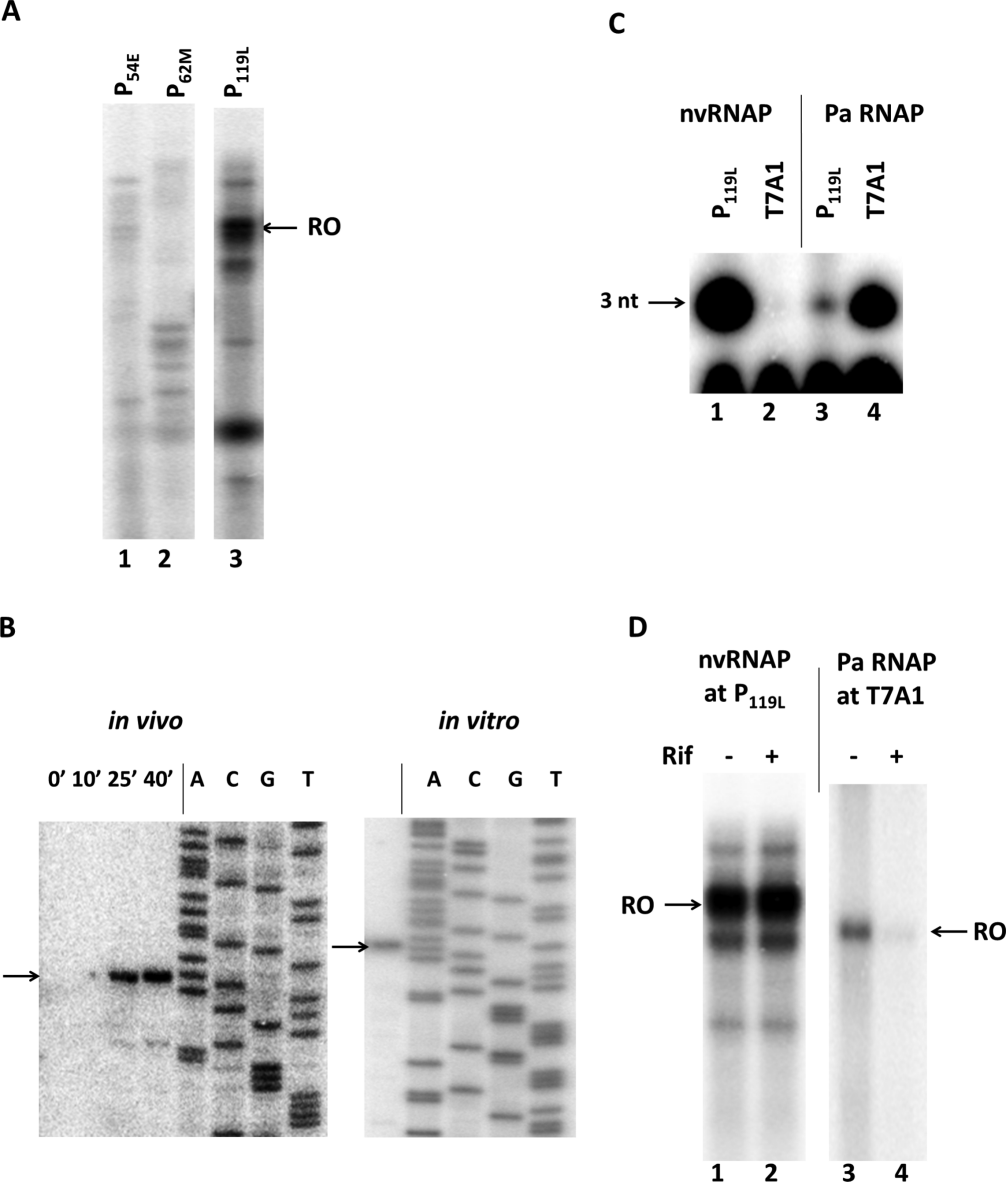
*In vitro* transcription by phiKZ nvRNAP. (**A**) Multi-round run-off *in vitro* transcription by nvRNAP from PCR fragments containing an early (P_54E_), middle (P_62M_) and late (P_119L_) phiKZ promoters. RO – run-off RNA products. (**B**) Primer extension mapping of 5′-ends of *in vivo* (left panel) and *in vitro* (right panel) transcripts from the phiKZ late promoter P_119L._ DNA sequencing reactions with the same end-labeled primer used as sequence markers are indicated. The arrows indicate primer extension products. (**C**) *In vitro* abortive initiation reactions by nvRNAP and host *P. aeruginosa* RNAP σ^70^-holoenzyme (Pa RNAP) from phiKZ late promoter (P_119L_) and bacterial RNAP promoter T7 A1. 3 nt – trinucleotide abortive transcripts. (**D**) *In vitro* run-off transcription by nvRNAP and *P. aeruginosa* RNAP from phiKZ P_119L_ and T7 A1 promoters in the presence or absence of rifampicin. RO – run-off RNA products.

Previously, early, middle and late phiKZ promoters were mapped by means of RNA-seq analysis and *in vivo* primer extension experiments with total RNA prepared from phiKZ-infected host cells collected at different times post-infection (30). In contrast to a well-conserved extended consensus element associated with early phage promoters, middle and late promoters were associated with weakly conserved short motifs (30). Since all subunits of purified phiKZ nvRNAP are the products of early genes, we expected that the enzyme should recognize middle and/or late phage promoters. To assess transcription activity and promoter specificity of the enzyme, the purified nvRNAP was tested in *in vitro* multiple-round run-off transcription assay using PCR-amplified DNA fragments containing an early, middle or late phage promoter as templates. Robust transcription was detected only in a reaction containing late P_119L_ promoter template (Figure 3A, lane 3).

Next, the *in vitro* transcription start points were mapped by primer extension. Mapped start points for P_119L_ (Figure 3B) and for every randomly chosen late phiKZ promoter tested (Supplementary Figure S2) coincided with corresponding late transcripts start points earlier determined *in vivo* (Figure 3B) (30). These start points were associated with a 5′-T^−3^ATG^+1–^3′ consensus motif. Thus, the purified five-subunit phiKZ nvRNAP is sufficient for specific recognition of and transcription from late phage promoters. The phiKZ nvRNAP was unable to transcribe from a strong σ^70^-depedent T7 A1 promoter that was recognized by host RNAP. Conversely, *P. aeruginosa* RNAP did not transcribe from a late phiKZ promoter (Figure 3C).

The phiKZ development does not depend on rifampicin, an inhibitor of bacterial RNAPs (30,37). As expected, the nvRNAP transcription from late promoter P_119L_ was insensitive to rifampicin, while control transcription by *P. aeruginosa* RNAP σ^70^ holoenzyme from a bacterial promoter was strongly inhibited (Figure 3D).

Тo further characterize nvRNAP complexes with late promoters we performed DNase I and KMnO_4_ footprinting, however, no detectable footprint was observed, suggesting that the promoter complexes are highly unstable. The instability of nvRNAP complexes with DNA is consistent with the fact that this enzyme is eluted from PEI pellets with buffers containing low concentration of salt (0.3 M NaCl), compared to 0.6–1.0 M concentration needed to elute host RNAP.

### The phiKZ late promoter conserved TATG motif is necessary but not sufficient for *in vitro* transcription by nvRNAP

**Figure 4.**
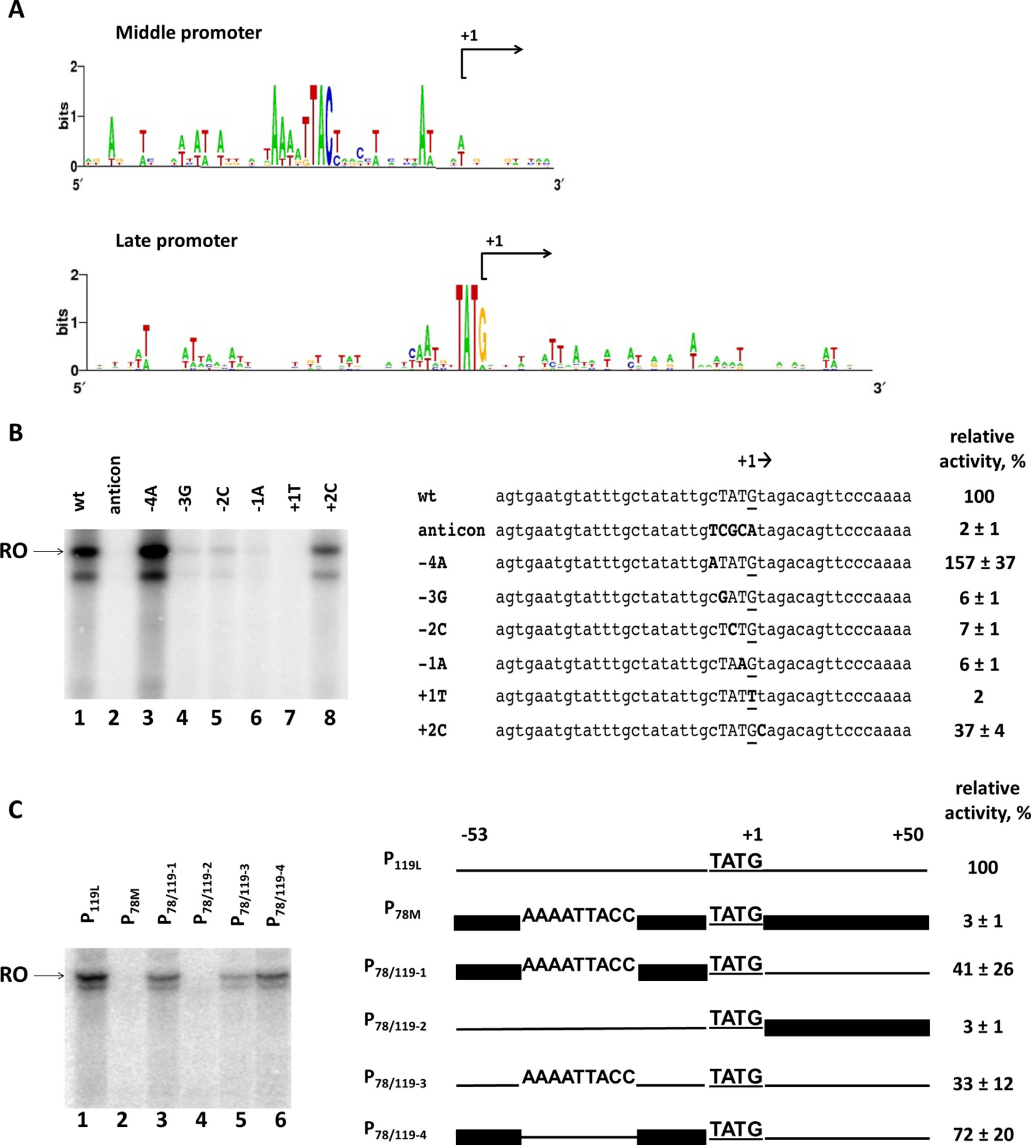
Promoter specificity determinants of nvRNAP. (**A**) Sequence logos of phiKZ middle and late promoters. (**B**) Analysis of the phiKZ late promoter consensus motif 5′-TATG-3′ by point mutations. Left panel – *in vitro* run-off transcription by nvRNAP from late promoter P_119L_ and its derivatives. RO – run-off RNA products. Right panel – an alignment of nucleotide sequences of wild-type and mutant promoters (introduced substitutions are shown in bold typeface). The +1 start site is underlined. (**C**) Mutational analysis of upstream and downstream sequences of phiKZ late promoter P_119L_. Left panel – *in vitro* run-off transcription by nvRNAP from chimeric templates based on phiKZ P_78M_ middle and P_119L_ late promoters. RO – run-off RNA products. Right panel – schematic representation of hybrid promoters used. The P_119L_ sequence is shown by a thin line; the P_78M_ is shown by a thick line. Numbers above the scheme indicate upstream (position −53 with respect to the start site) and downstream (position +51) boundaries of promoter fragments used. The late consensus TATG motif with the +1 start of transcription is underlined. The putative middle promoter conserved motif 5′-AAAATTACC-3′ is also indicated. The numbers at the right indicate the transcription activities relative to the positive control (wild-type P_119L_). Average values and standard deviations from three independent experiments are presented.

The phiKZ late promoters are associated with a consensus 5′-TATG-3′ motif, with the last nucleotide being a transcription start site (Figure 4A). To investigate whether this motif is necessary for nvRNAP transcription we systematically replaced each of the four consensus positions of the late phiKZ promoter P_119L_ and tested the resulting mutant templates in *in vitro* multiple-round run-off transcription assay. In addition, an anticonsensus derivative with all four positions of the motif replaced was tested. Every single substitution at the consensus motif led to dramatic reduction of nvRNAP transcription (Figure 4B, lanes 4–7). In contrast, substitutions outside the consensus, at positions −4 and +2, either had a smaller inhibitory effect or even stimulated nvRNAP transcription (Figure 4B, lanes 3 and 8). No transcription from P_119L_ anticonsensus derivative was detected (Figure 4B, lane 2). We conclude that the late promoter 5′-TATG-3′ conserved motif is necessary for *in vitro* transcription by nvRNAP.

The high specificity of nvRNAP to late phage promoters is somewhat puzzling since 5′-TATG-3′ sequences occur with high frequency in the phiKZ genome. In particular, while phiKZ middle promoters have a specific motif 5′-AAanTTACc-3′ centered ca. position −24 with respect to the transcription start site, two middle promoters, P_62M_ and P_78M_, also have appropriately positioned late TATG motif and yet are not efficiently recognized by nvRNAP *in vitro* (Supplementary Figure S3). To determine the possible contribution of upstream and downstream promoter sequences to promoter selection by nvRNAP, we constructed a series of hybrid templates based on sequences of middle promoter P_78M_ and late promoter P_119L_. The first hybrid template, P_78/119–1_, contains the P_78M_ sequence upstream of the +1 position and the P_119L_ downstream sequence. The second, reciprocal hybrid, P_78/119–2_, contains the upstream sequence from P_119L_ and the P_78M_ downstream sequence. The third derivative, P_78/119–3_, contains the putative middle promoter conserved motif 5′-AAAATTACC-3′ from P_78M_ instead of the corresponding sequence of P_119L_. The final construct, P_78/119–4_, is a derivative of the P_78/119–1_ template, in which the conserved middle promoter element of P_78M_ is replaced with the corresponding sequence from P_119L_ (Figure 4C). When hybrid templates were tested in *in vitro* multiple-round run-off transcription assay, significant transcription was only detected from templates containing the P_119L_ downstream sequence (Figure 4C, lanes 1, 3, 5 and 6). The introduction of the 5′-AAAATTACC-3′ middle promoter motif into P_119L_ decreased transcription 3-fold. Removal of this element from a hybrid template containing the P_78M_ upstream sequence also increased transcription. Thus, the middle promoter element appears to negatively modulate late promoter recognition by nvRNAP, though it is clearly not the only element preventing the recognition of middle promoters with late TATG motif. The downstream portion of the P_119L_ promoter DNA apparently contains the primary sequence element(s) required for efficient transcription from TATG motif-containing promoters: substitution of this segment with P_78M_ DNA results in ∼30-fold reduction of transcription. However, no additional sequence conservation downstream of the TATG motif could be detected in phiKZ late promoters sequences (Figure 4A).

## DISCUSSION

In this work we purified a novel multisubunit RNAP from phiKZ-phage infected *P. aeruginosa* cells. The purified enzyme, non-virion phiKZ RNAP, consists of two pairs of polypeptides with proteins from each pair together roughly corresponding to second largest (β) and largest (β’) catalytic subunits of cellular RNAPs. The fifth subunit, gp68, does not have sequence similarity with any known protein. The phiKZ nvRNAP subunits do not have identifiable sequence similarity to msRNAP assembly subunits (such as α and ω in bacterial RNAPs) or initiation factors such as σ or general transcription factors of archaeal or eukaryal RNAPs responsible for promoter recognition and transcription initiation. Yet, nvRNAP is able to efficiently recognize and transcribe from phage late promoters *in vitro* with *in vivo* specificity. Homologs of gp68 are found in all phiKZ group phages, albeit they are more divergent than other RNAP subunits encoded by these phages. It remains to be seen whether gp68 is responsible for promoter recognition, nvRNAP subunit assembly, or both. Be that as it may, the conserved late phiKZ promoter motif required for nvRNAP transcription is located immediately upstream of the transcription start point, which is in contrast to the −10 promoter consensus element or the TATA box location in, correspondingly, bacterial or eukaryal promoters. So, irrespective of which nvRNAP subunit is responsible for promoter recognition and melting, the mechanism is likely to be novel.

The phiKZ nvRNAP recognizes a late promoter consensus 5′-TATG-3′ that overlaps with transcription start point at the 3′-terminal guanosine. While substitutions in the consensus motif abolish or strongly reduce transcription other, yet-to-be-defined sequences are also needed to form a functional late phage promoter. Functional and structural work currently in progress in our laboratory will illuminate the mechanisms of promoter recognition by nvRNAP and allow comparisons with bacterial and eukaryal systems.

An unusual multisubunit RNAP has been previously isolated from *B. subtilis* infected with PBS2, a large general transducing phage containing deoxyuracil instead of thymine in its DNA (27,28). The sequence of PBS2 genome is not known at this time and so is its relationship, if any, to the phiKZ-like phages cannot be ascertained. It is worth noting, however, that the giant *Yersinia* phage phiR1–37 also contains deoxyuracyl in its DNA (38). The PBS2 RNAP consists of five subunits whose apparent molecular weights roughly match the molecular weights of phiKZ nvRNAP. Also like phiKZ nvRNAP, the PBS2 enzyme is rifampicin-resistant and transcribes late phage genes (27). Interestingly, the PBS2 enzyme was reported to be purified in two forms from infected cells: a five-subunit complex and a four-subunit complex lacking the P53 subunit (27,28). The phiKZ gp68, the only nvRNAP subunit with no identifiable similarity to any known RNAP subunits, is tightly associated with the rest of nvRNAP subunits and we were unable to separate it from the complex. The relationship, if any, between the PBS2 and phiKZ nvRNAPs will become apparent once the sequence of the *B. subtilis* phage genome is determined.

With the exception of PBS2, bacteriophage phiKZ is the only known dsDNA phage that is independent of host RNAP transcription. Two phage-encoded non-canonical msRNAPs appear to transcribe phage genes throughout phiKZ development using promoters of three temporal classes. The nvRNAP purified in this work is composed of product of early phage genes and recognizes promoters of late phage genes. Given that middle and late transcripts have different kinetics and the respective phage promoters have clearly distinct consensus motifs (30), at least two scenarios of middle phiKZ transcription can be considered. First, vRNAP may be modified by a phage early protein to recognize middle promoters. Second, nvRNAP may recognize both classes of promoters, middle and late, upon modification by early and/or middle phage protein, respectively. It is also possible that promoter specificity switch is accomplished by a phage-encoded DNA-binding protein(s) that recruits one of the two RNAPs to middle promoters. Purification of phiKZ vRNAP and/or possible alternative forms of nvRNAP will be needed to resolve this interesting question.

## Supplementary Material

SUPPLEMENTARY DATA
