# Supplementary material for: A non-canonical multisubunit RNA polymerase encoded by a giant bacteriophage
Source: Nucleic Acids Res. 2015 Oct 20;43(21):10411–20. doi: 10.1093/nar/gkv1095 (PMC4666361; doi:10.1093/nar/gkv1095)
Supplement: SUPPLEMENTARY DATA [file supp_43_21_10411__index.html]

A non-canonical multisubunit RNA polymerase encoded by a giant bacteriophage — SUPPLEMENTARY DATA 

# A non-canonical multisubunit RNA polymerase encoded by a giant bacteriophage

## SUPPLEMENTARY DATA

- SUPPLEMENTARY DATA
