# Supplementary material for: A non-canonical multisubunit RNA polymerase encoded by a giant bacteriophage
Source: Nucleic Acids Res. 2015 Oct 20;43(21):10411–20. doi: 10.1093/nar/gkv1095 (PMC4666361; doi:10.1093/nar/gkv1095)
Supplement: SUPPLEMENTARY DATA [file supp_gkv1095_nar-02209-v-2015-File006.doc]

**Supplementary material**

**A. Second largest (β-like) subunits**

structural features

A = Beta1

B = Beta2

C = Beta-flap

F = Switch3

G = Clamp (there is also an rpoC part)


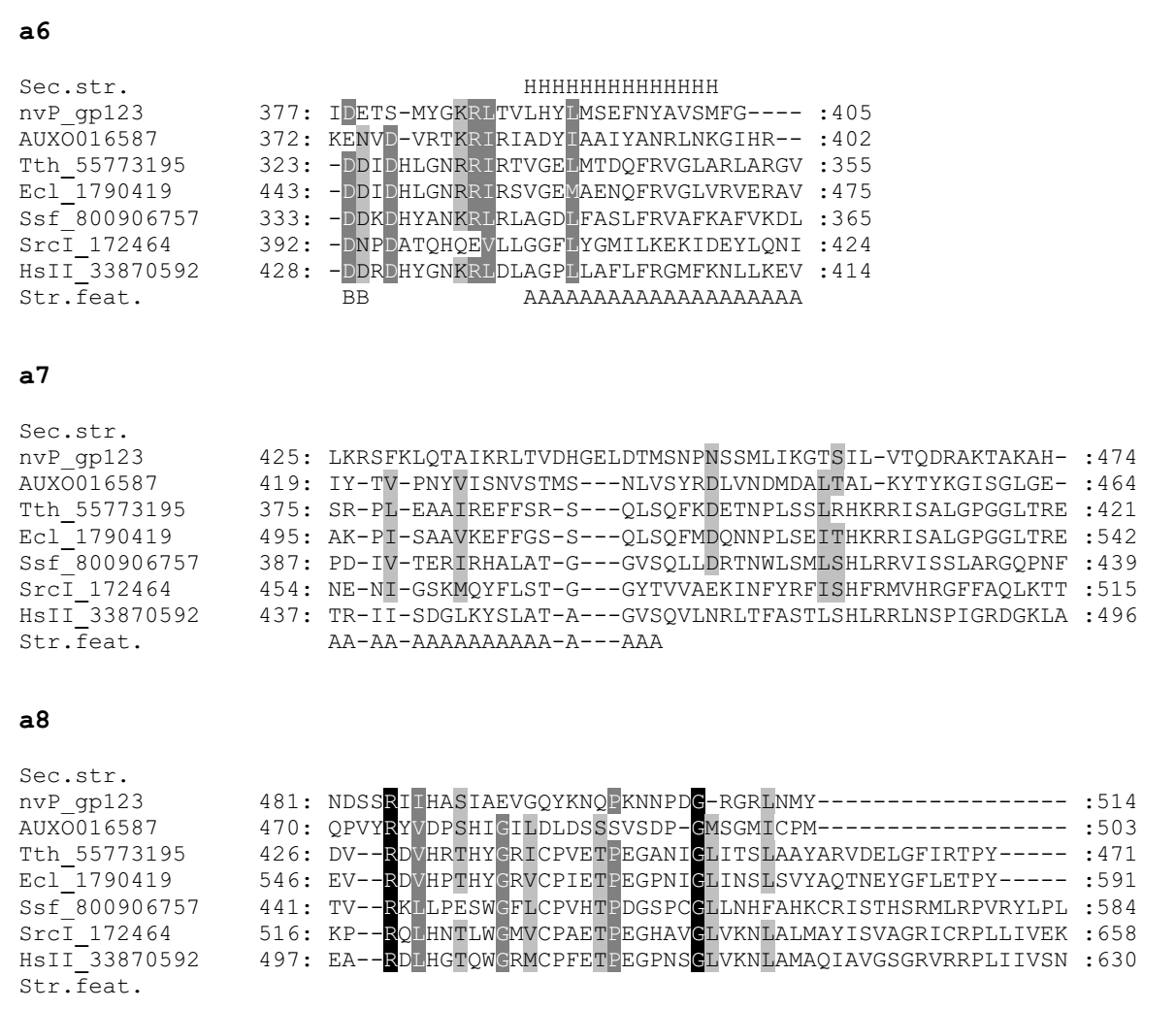


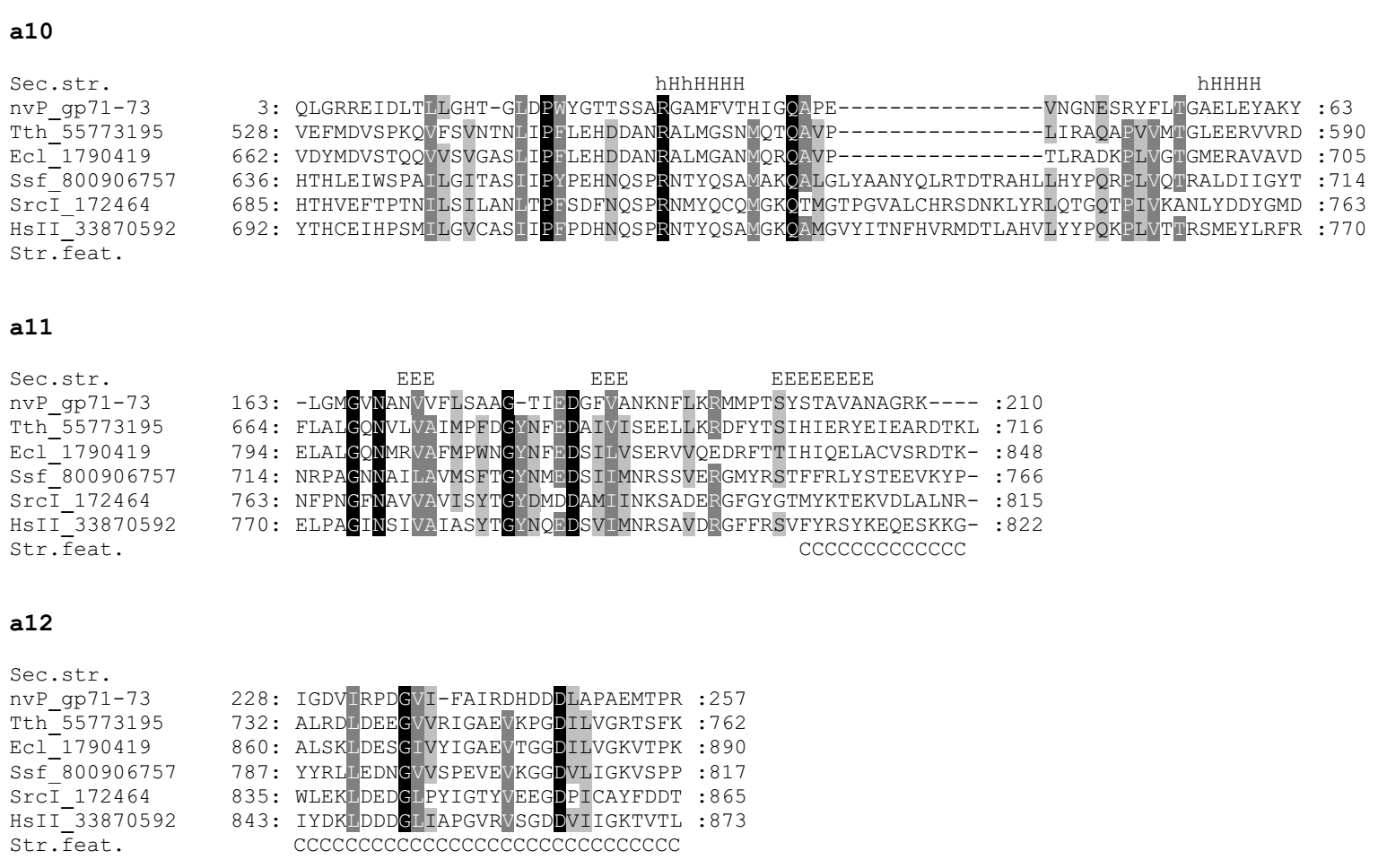


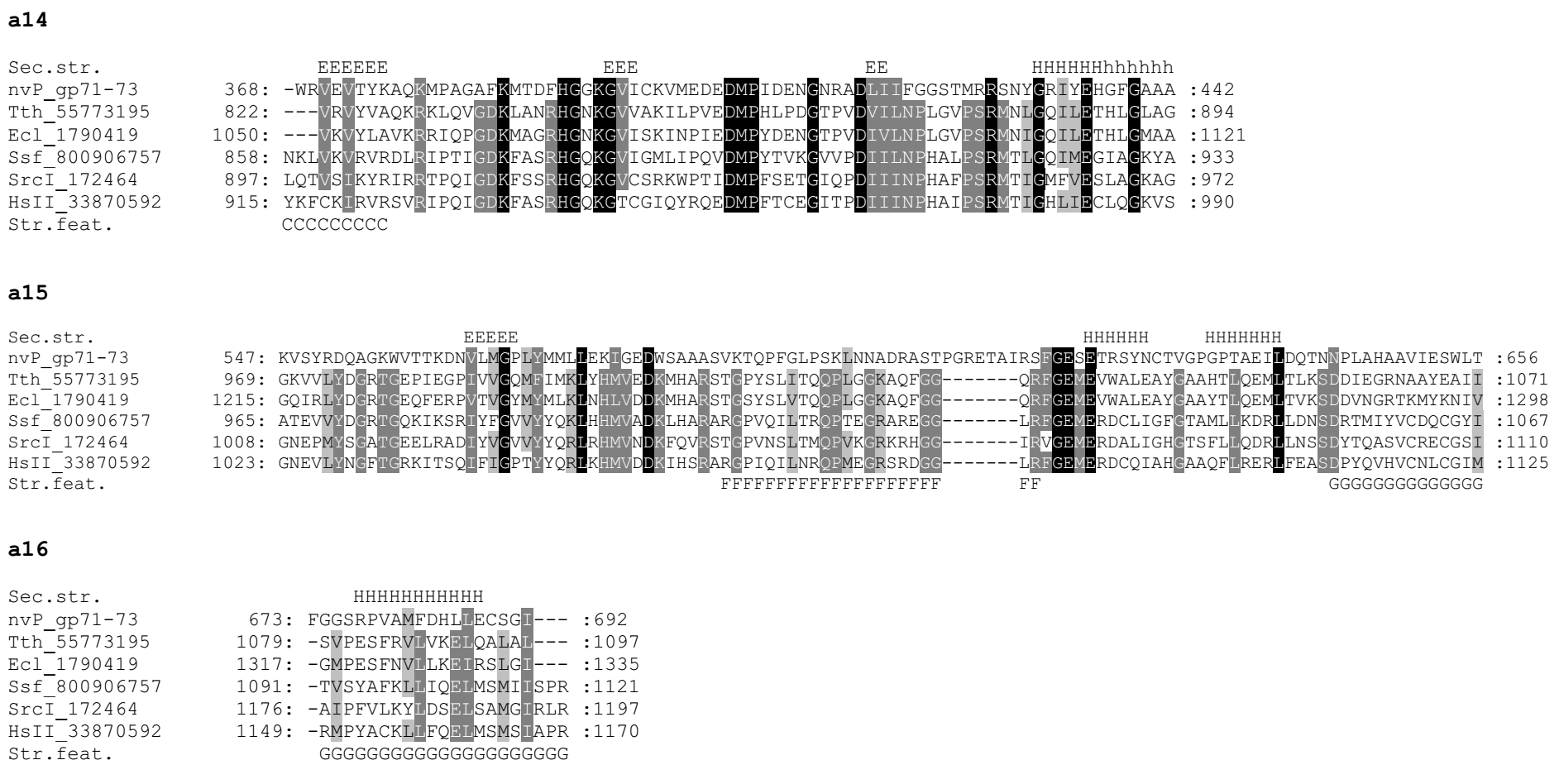


**B. **  **Largest (β’–like) subunits**

Structural features:

G = Clamp (there is also an rpoB part)

I = Lid (includes Clamp)

J = Beta_Prime-coiled-coil (includes Clamp)

K = Rudder (includes Clamp and beta-prime coiled-coil)

L = Switch2

M = Secondary-channel_rim_helices

N = Bridge_helix

O = Trigger_loop_helix1

P = Trigger_loop

| 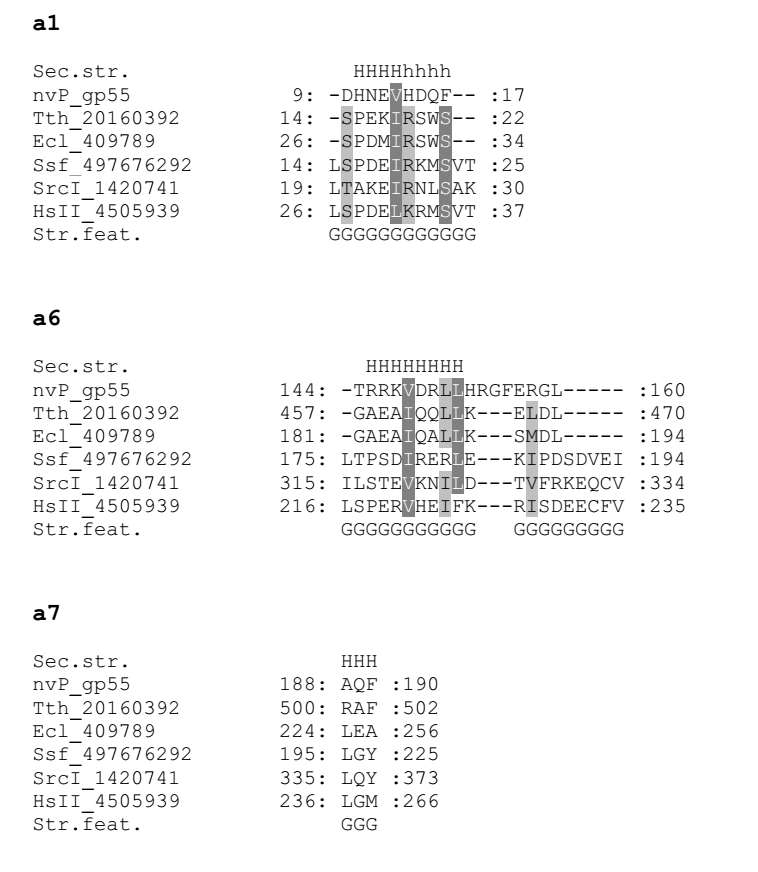 | 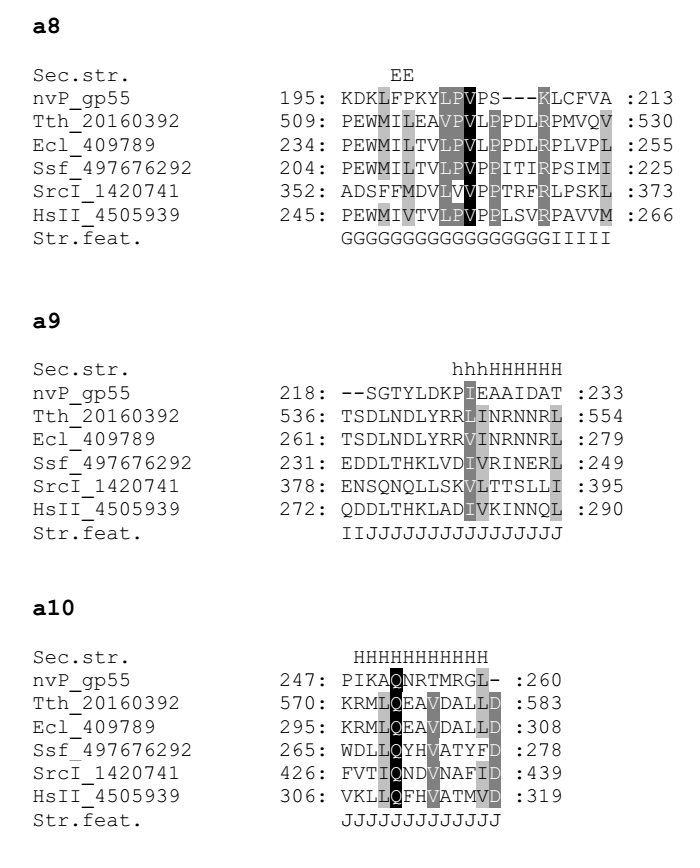 |
| --- | --- |


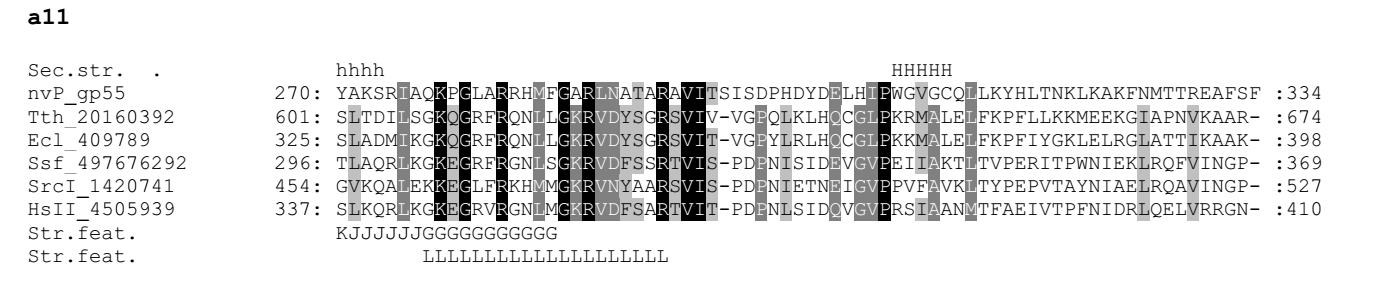


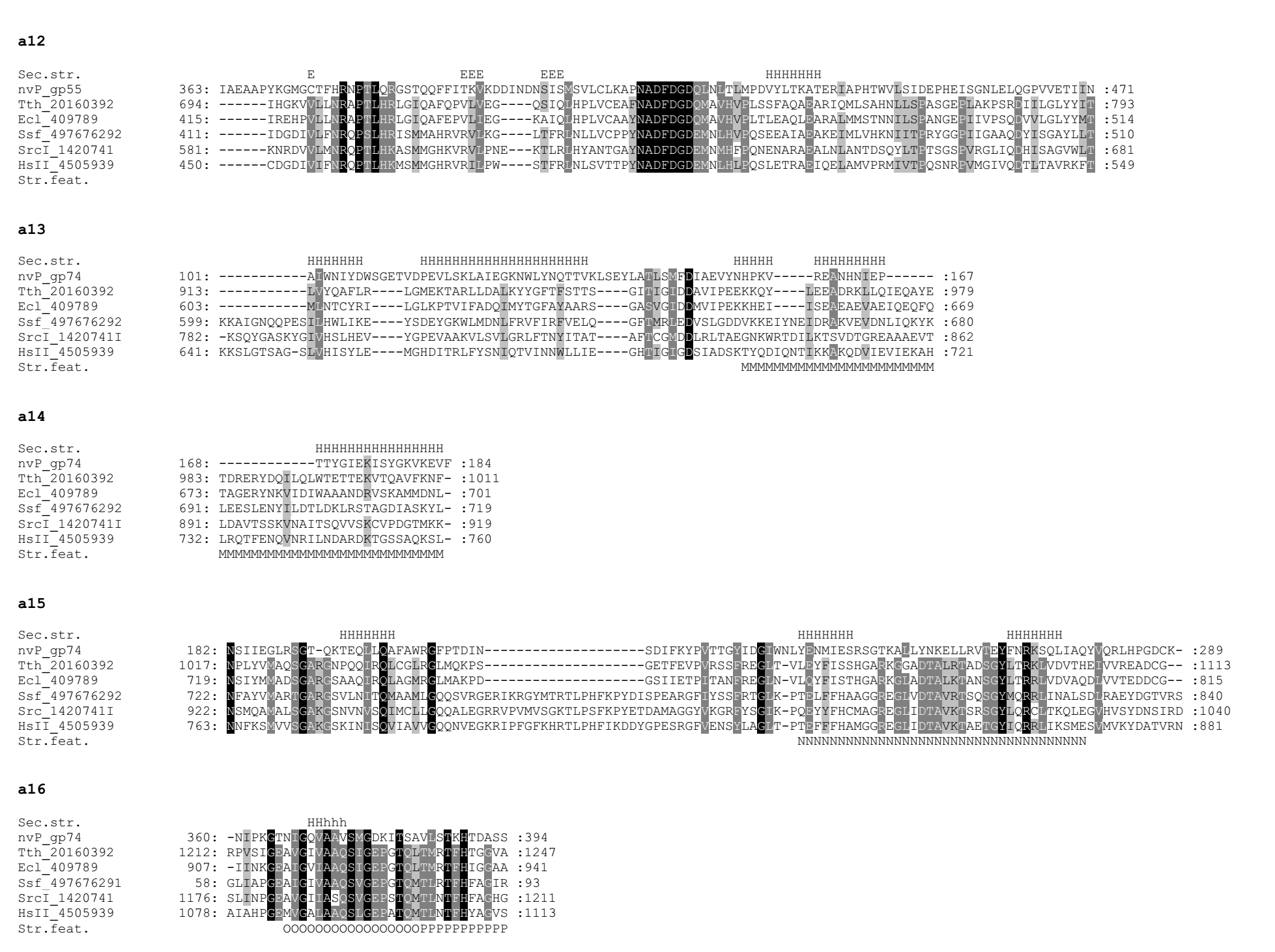


**Figure S1. Multiple sequence alignments of msRNAP conserved regions identified in phiKZ nvRNAP subunit sequences*.*** Alignments of phiKZ nvRNAP subunit sequences with second largest/β (**А**) and largest/β’ (**B**) msRNAP subunits conserved regions are presented. In the beginning of each set of alignments designations of msRNAP structural features are listed according to Lane and Darst (1,2), with each feature labeled with letters. These structural features are indicated below the alignments with same letters. The predicted secondary structure elements are shown above the alignments: H - helices, E –strands. The positions of the first and the last residue of aligned regions in proteins used to build the alignment are indicated, respectively, before and after each sequence. The figure was prepared using GenDoc. The shadows of grey indicate the extent of conservation. The sequences of phiKZ nvRNAP subunits are denoted by protein names. The sequences of canonical msRNAP subunits are denoted by abbreviated names and Gene Identification (GI) numbers from GenBank database. Abbreviations used: nvP – phiKZ nvRNAP; Tth – *Thermus thermophilus* RNAP β/β’, Ecl – *Escherichia coli* RNAP β/β’, Ssf – *Sulfolobus solfataricus* RNAP rpoB’-B”/A’-A”, SrcI – *Saccharomyces cerevisiae* PolI RPA190/135, HsII – *Homo sapiens* PolII RPB1/2. In the case of gp123 we were not able to identify regions similar to msRNAP subunit conserved regions directly. However, a BLAST search against WGS database using a gp123 homolog from Yersinia_phage_phiR1-37, gp105, identifies multiple hits including a protein gb|AUXO016587845.1| from gut metagenome contig-1047000524 with an E-value=10e-35. This protein (labeled AUXO016587) gives a good alignment with the N-terminal half of bacterial msRNAP β subunits (>90% HHpred probability). The alignment covers conserved regions a6, a7 and a8, indicating that these proteins are homologs of the N-terminal part of β subunits. Thus, gp123 was added to HHpred alignment of AUXO016587845.1 to msRNAP subunits.


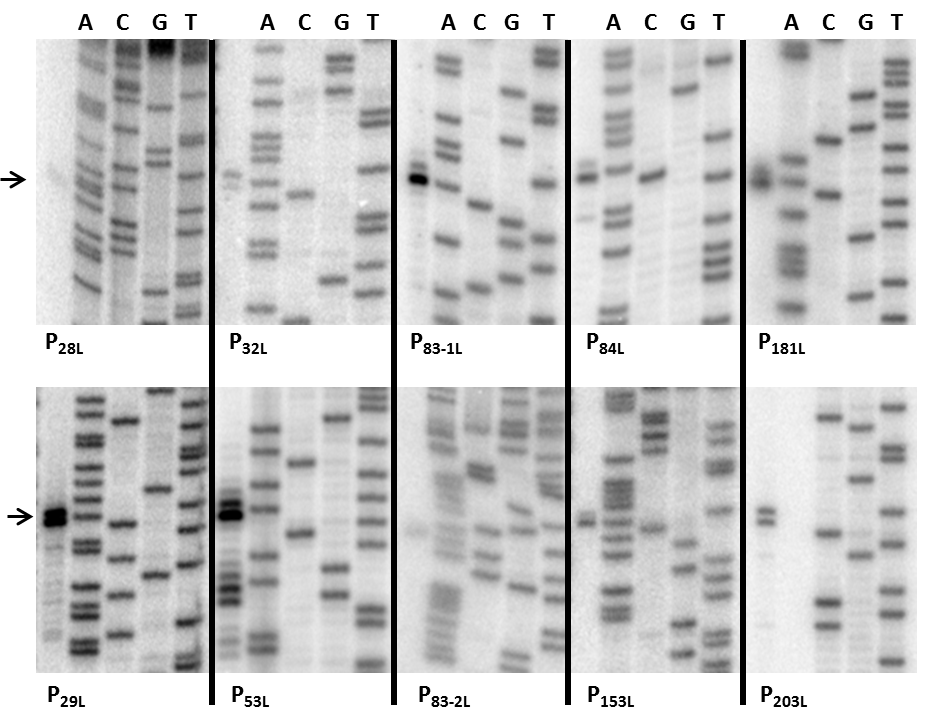


**Figure S2. Mapping of phiKZ late promoters *in vitro.*** The results of *In vitro* transcription by nvRNAP from DNA fragments containing ten late phiKZ promoters followed by primer extension reaction are shown. Arrows indicate the primer extension products. DNA sequencing reactions with the respective end-labeled primers used as sequence markers are indicated. All *in vitro* established starts of transcription by nvRNAP match those revealed *in vivo.* There are two late promoters upstream of phiKZ gene 83, P_83-1L_ and P_83-2L_. Analysis of the tenth P_119L_ primer extension product is given in the Fig. 3B


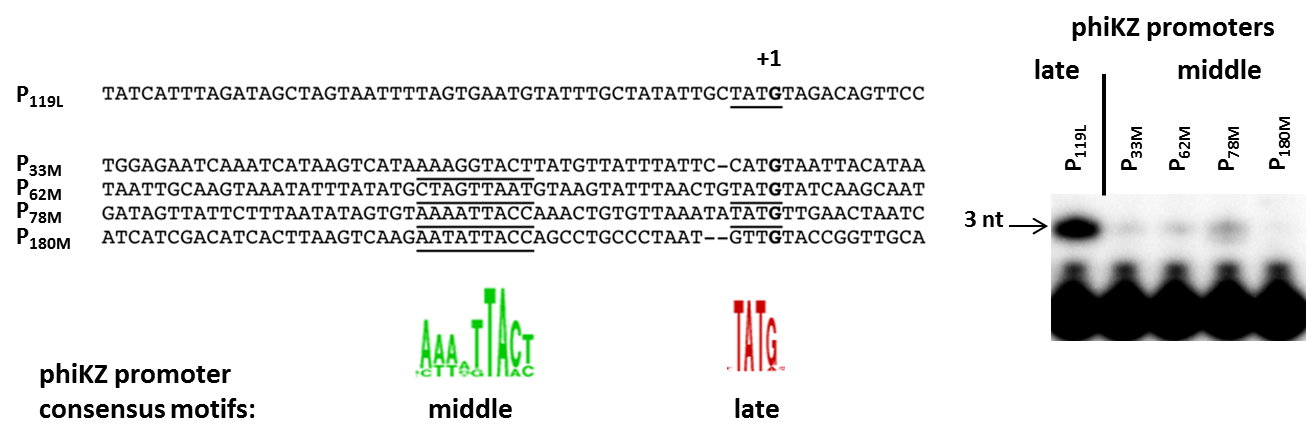


**Figure S3. The nvRNAP does not transcribe from phiKZ middle promoters *in vitro.*** Right panel – *in vitro* abortive initiation by nvRNAP from phiKZ late promoter P_119L_ and selected middle promoters P_33M_, P_62M_, P_78M_ and P_180M_. Left panel – an alignment of sequences of these promoters. Putative middle and established late consensus elements are underlined in the aligned sequences; transcription start sites are shown in bold. Below the alignment the corresponding middle and late promoter logos are indicated.

REFERENCES

1. Lane, W.J. and Darst, S.A. (2010) Molecular evolution of multisubunit RNA polymerases: sequence analysis. *J Mol Biol*, **395**, 671-685.

2. Lane, W.J. and Darst, S.A. (2010) Molecular evolution of multisubunit RNA polymerases: structural analysis. *J Mol Biol*, **395**, 686-704.
